# Benthic ammonia oxidizers differ in community structure and biogeochemical potential across a riverine delta

**DOI:** 10.3389/fmicb.2014.00743

**Published:** 2015-01-08

**Authors:** Julian Damashek, Jason M. Smith, Annika C. Mosier, Christopher A. Francis

**Affiliations:** Department of Environmental Earth System Science, Stanford UniversityStanford, CA, USA

**Keywords:** nitrification, archaea, bacteria, amoA, estuary, river, salinity, ammonium

## Abstract

Nitrogen pollution in coastal zones is a widespread issue, particularly in ecosystems with urban or agricultural watersheds. California's Sacramento-San Joaquin Delta, at the landward reaches of San Francisco Bay, is highly impacted by both agricultural runoff and sewage effluent, leading to chronically high nutrient loadings. In particular, the extensive discharge of ammonium into the Sacramento River has altered this ecosystem by vastly increasing ammonium concentrations and thus changing the stoichiometry of inorganic nitrogen stocks, with potential effects throughout the food web. This debate surrounding ammonium inputs highlights the importance of understanding the rates of, and controls on, nitrogen (N) cycling processes across the delta. To date, however, there has been little research examining N biogeochemistry or N-cycling microbial communities in this system. We report the first data on benthic ammonia-oxidizing microbial communities and potential nitrification rates for the Sacramento-San Joaquin Delta, focusing on the functional gene *amoA* (which codes for the α-subunit of ammonia monooxygenase). There were stark regional differences in ammonia-oxidizing communities, with ammonia-oxidizing bacteria (AOB) outnumbering ammonia-oxidizing archaea (AOA) only in the ammonium-rich Sacramento River. High potential nitrification rates in the Sacramento River suggested these communities may be capable of oxidizing significant amounts of ammonium, compared to the San Joaquin River and the upper reaches of San Francisco Bay. Gene diversity also showed regional patterns, as well as phylogenetically unique ammonia oxidizers in the Sacramento River. The benthic ammonia oxidizers in this nutrient-rich aquatic ecosystem may be important players in its overall nutrient cycling, and their community structure and biogeochemical function appear related to nutrient loadings. Unraveling the microbial ecology and biogeochemistry of N cycling pathways, including benthic nitrification, is a critical step toward understanding how such ecosystems respond to the changing environmental conditions wrought by human development and climate change.

## Introduction

In the past century, widespread synthetic ammonia production has profoundly perturbed the global nitrogen (N) cycle (Erisman et al., 2008). Industrial N fixation rates are now comparable to or even greater than natural sources, leading to a doubling of the planet's fixed N inventory (Galloway et al., 2004; Billen et al., 2013; Erisman et al., 2013). Local ecological effects of this global phenomenon have been drastic. In particular, agricultural runoff and sewage treatment effluent can greatly enrich aquatic habitats with respect to N, often stimulating primary production and leading to eutrophication (Erisman et al., 2013), the consequences of which can include hypoxia (Rabalais et al., 2010) and harmful algal blooms (Anderson et al., 2002), among others. However, a substantial fraction of fixed N inputs to rivers and estuaries is removed *in situ* by two microbial pathways: denitrification and the anaerobic oxidation of ammonia (anammox; Boynton and Kemp, 2008; Ward, 2013). In well-mixed rivers and estuaries, these N loss pathways are typically active only within anoxic sediments, and ultimately provide an ecological N sink by converting fixed N to nitrogen gas (N_2_). In this way, microbes provide a tremendous “ecosystem service” by shuttling N out of aquatic ecosystems and back to the atmosphere.

The N cycle is a set of coupled reactions that transform N from one compound into another (Ward, 2012). One particularly important component is nitrification, the microbially-catalyzed conversion of ammonia (NH_3_) to nitrate (NO^−^_3_). This process occurs in two steps: (1) ammonia oxidation, typically the rate-limiting step, converts NH_3_ to nitrite (NO^−^_2_); and (2) nitrite oxidation further converts NO^−^_2_ to NO^−^_3_. Importantly, NO^−^_3_ and NO^−^_2_ are substrates for denitrification and anammox. Since nitrification is the only process that transforms remineralized ammonium (NH^+^_4_) into the inorganic forms that feed N loss, it is the crucial link between fixed N inputs and outputs in an ecosystem. Therefore, understanding environmental controls on the microbial ecology and biogeochemistry of ammonia oxidation is critical for understanding how N is cycled through, and ultimately lost from, any aquatic ecosystem.

For over a century, it was believed the capacity for ammonia oxidation was confined to the phylum *Proteobacteria* (Kowalchuk and Stephen, 2001). Recently, the discovery of ammonia-oxidizing archaea (AOA; Könneke et al., 2005), now known as “Thaumarchaeota” (Brochier-Armanet et al., 2008), made this story more complex. AOA are present in many environments (Francis et al., 2005; Treusch et al., 2005; Beman et al., 2007; Spear et al., 2007; De Corte et al., 2008; Biller et al., 2012), frequently outnumber AOB in marine and terrestrial systems (Leininger et al., 2006; Wuchter et al., 2006; Beman et al., 2008; Weidler et al., 2008; Santoro et al., 2010; Nicol et al., 2011), and appear to drive nitrification rates throughout the marine water column (Wuchter et al., 2006; Beman et al., 2012; Newell et al., 2013; Smith et al., 2014a). However, the complexity of ammonia-oxidizing communities in estuaries has precluded efforts to uncover broad estuarine trends. Some estuaries are dominated by AOA (Beman and Francis, 2006; Caffrey et al., 2007; Moin et al., 2009), others by AOB (Abell et al., 2010; Bernhard et al., 2010), and some have alternating zones of AOA and AOB dominance (Mosier and Francis, 2008; Santoro et al., 2008; Bouskill et al., 2012a; Zheng et al., 2014). Additionally, studies of ammonia oxidizers in freshwater ecosystems are relatively rare, and rivers are particularly understudied (Biller et al., 2012), though the handful of studies published to date have indicated the ammonia-oxidizing communities in river sediments are often quite complex (Liu et al., 2013; Sonthiphand et al., 2013; Wang et al., 2014).

**Figure 1:**
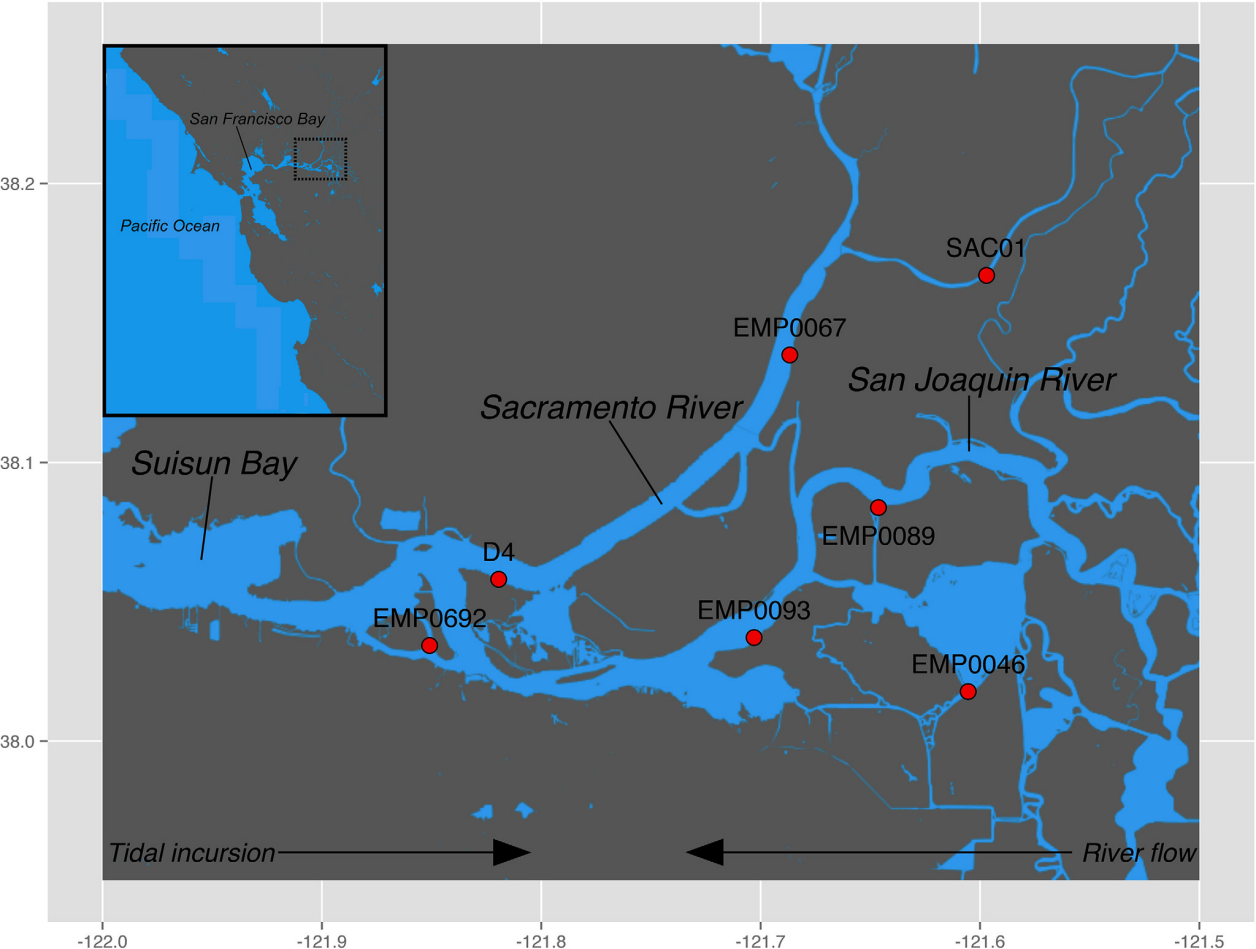
**Sampling locations in the Sacramento-San Joaquin Delta**. Regions sampled were the Sacramento River (stations SAC01 and EMP0067), the San Joaquin River (stations EMP0089 and EMP0093), Franks Tract (station EMP0046), and the upper oligohaline region of the estuary (San Francisco Bay) at the confluence of the two rivers (stations D4 and EMP0692). Generally, river flow moves westward while tides propagate eastward. Inset shows the greater region of San Francisco Bay, with the dotted black box denoting the Delta.

The Sacramento-San Joaquin River Delta (“the Delta”) is a network of tidally influenced freshwater marshes, rivers, and islands where the Sacramento and San Joaquin rivers meet San Francisco Bay (Figure 1). The Delta drains a 153,000 km^2^ watershed encompassing 40% of the area of California, including the entire Central Valley (Conomos et al., 1985), and receives high nutrient loads from agricultural runoff and wastewater effluent (Cloern and Jassby, 2012). In the past two centuries, anthropogenic development throughout California has led to massive alterations within the Delta: huge swaths of wetlands have been diked and drained to create arable farmland, massive volumes of water are pumped south for agricultural and urban use, and increased wastewater discharge has elevated nutrient concentrations (Lund et al., 2010). The recent collapse of numerous fish populations, termed the “pelagic organism decline” (Sommer et al., 2007), has focused considerable scientific and political attention on water quality issues in the Delta. While the piscine population crashes appear to be caused by a complex array of environmental forces (MacNally et al., 2010; Thomson et al., 2010), two potential stressors have come under particular scrutiny, due to their implications for management: freshwater exports from the Delta (Kimmerer, 2008), and the massive discharge of NH^+^_4_ into the Sacramento River by the Sacramento Regional Wastewater Treatment Plant (Glibert, 2010; Glibert et al., 2011). While this debate highlights the importance of understanding the sources, sinks, and transformation rates of NH^+^_4_ in the Delta, little is known about a key aspect of the N cycle in this system: benthic nitrification and the associated ammonia-oxidizing microbial assemblages. A recent study measuring rates of benthic N_2_, NH^+^_4_, and NO^−^_3_ fluxes across northern San Francisco Bay and the Delta showed high but seasonally-variable rates of coupled nitrification-denitrification, with high rates reported from Delta sediments, compared to the brackish sediments further downstream (Cornwell et al., 2014). Therefore, benthic nitrification may be an important driver of N loss in the Delta. Here, we assess the diversity, abundance, and biogeochemical potential of benthic ammonia-oxidizing communities throughout the Delta. This work is the first analysis of ammonia-oxidizing communities and benthic potential nitrification rates in this ecosystem.

## Materials and methods

### Sediment and bottom water data collection

Sampling stations were selected to encompass both riverine systems (Sacramento, San Joaquin) as well as the confluence of the rivers in the “upper estuary” oligohaline region of San Francisco Bay. Additionally, one station was located in Franks Tract, a slowly flushed tidal lake adjacent to the San Joaquin river (Figure 1). Samples were collected in September and October 2007 onboard the R/V *Endeavor*. Surface sediment was retrieved using a modified Van Veen grab. Duplicate cores were taken from each grab sample using sterile, cut-off 5 mL syringes and immediately placed on dry ice prior to storage at −80°C. Bottom water nutrient samples were collected it duplicate using a hand-held Niskin bottle, immediately filtered (0.2 μm pore size), and frozen on dry ice prior to storage at −20°C. Nutrient (NH^+^_4_, NO^−^_2_, and NO^−^_3_) concentrations were measured using a QuikChem 8000 Flow Injection Analyzer (Lachat Instruments). Bottom water alkalinity, conductivity, dissolved oxygen, pH, and temperature data were collected by the California Water Resources Control Board's sediment quality objectives program, in collaboration with the Department of Water Resources, and were provided by the Southern California Coastal Water Research Project (Steve Bay, personal communication). Dayflow data were downloaded from the California Department of Water Resources database (http://www.water.ca.gov/dayflow/).

### Potential nitrification rates

Sediment samples for potential nitrification rate measurements were collected in triplicate into the barrels of cut-off 60 mL syringes, which were sealed with parafilm and transported to the laboratory on ice. Potential rates were measured using amended sediment slurries (Hansen et al., 1981; Henriksen et al., 1981). Slurries included 5 g of sediment (top 2 cm) homogenized in 50 mL artificial seawater (adjusted to site-specific salinities) augmented with a final concentration of 500 μM ammonium sulfate. Amended slurries were shaken (200 rpm) in the dark for 24 h at room temperature (~22°C). Aliquots for the determination of NO^−^_3_ plus NO^−^_2_ (NO_X_) were collected at evenly spaced intervals through the incubation period and stored at −20°C. Prior to analysis, aliquots were thawed and passed through Whatman No. 42 filter paper, and the filtrate was analyzed for the accumulation of NO_X_ over time, using a SmartChem 200 Discrete Analyzer (Unity Scientific). Rates were determined by linear regression of NO_X_ concentrations over time.

### DNA extraction and functional gene analyses

DNA was extracted from approximately 0.5 g of surface sediments by extruding and cutting the top 0.5 cm from frozen cores with a sterile scalpel and immediately proceeding with the FastDNA SPIN Kit for Soil (MP Biomedicals), including a bead beating step of 30 s at speed 5.5. All DNA extracts were visually checked by gel electrophoresis and quantified using the Qubit dsDNA BR assay (Life Technologies).

AOA and AOB *amoA* genes were quantified using gene-specific SYBR qPCR assays on a StepOnePlus Real-Time PCR System (Life Technologies). AOA *amoA* reactions contained iTaq SYBR Green Supermix with ROX (Bio-Rad Laboratories), 0.4 μM primers Arch-amoAF/Arch-amoAR (Francis et al., 2005) and 1 μL template DNA. AOA qPCR program details were identical to previously published protocols (Mosier and Francis, 2008) but with a 10 s detection step at 78.5°C. AOB *amoA* qPCR reactions used primers amoA1F/amoA2R (Rotthauwe et al., 1997), and were set up following Mosier and Francis (2008) but with a 10 s detection step at 83°C. Each plate included a standard curve (5–10^6^ copies/reaction) made by serial dilution of linearized plasmids extracted from previously sequenced clones, and negative controls that substituted sterile water for DNA. All standard curves had *R*^2^ ≥ 0.99, and reaction efficiency ranged from 90.9 to 94.3% (AOA) and 86.0 to 88.3% (AOB). Specificity was determined using melt curves. Data were discarded if the standard deviation between triplicate reactions was >15%; occasionally, obvious outliers were excluded and the midpoint of duplicate values was used.

The diversity of ammonia oxidizing communities was determined by cloning and sequencing of PCR-amplified *amoA* genes using primers Arch-amoAF/Arch-amoAR (Francis et al., 2005) and amoA1F*/amoA2R (Rotthauwe et al., 1997; Stephen et al., 1999) for AOA and AOB, respectively. Reaction conditions and PCR programs followed previously published protocols (Mosier and Francis, 2008). Triplicate reactions were qualitatively checked by gel electrophoresis, pooled, and purified using the MinElute PCR Purification Kit or MinElute Gel Extraction Kit (Qiagen), following the manufacturer's instructions. Purified products were cloned using the pGEM-T Vector System II (Promega), and sequenced by Elim Biopharmaceuticals on a 3730xl capillary sequencer (Life Technologies), using the M13R primer. Sequences were imported into Geneious (version 6.1.6 created by Biomatters, available from http://www.geneious.com) and manually cleaned prior to operational taxonomic unit (OTU) grouping (≥95% sequence similarity) using mothur (Schloss et al., 2009). Rarefaction curves and diversity/richness estimators (Chao1 and Shannon indices) were calculated using mothur. OTUs were aligned with reference sequences using the MUSCLE alignment package within Geneious, using a gap open score of −750. Alignments were manually checked and used to build neighbor-joining bootstrap trees (Jukes-Cantor distance model, 1000 neighbor joining bootstrap replicates) within Geneious. The *amoA* sequences generated in this study have been deposited into GenBank with accession numbers KM000240–KM000508 (AOB) and KM000509–KM000784 (AOA).

### Statistical analyses

Due to the low number of samples (*n* = 7), standard statistical analyses such as *t*-tests and ANOVAs could not be used to compare environmental data between stations or regions, due to a lack of statistical power. Two-tailed Spearman rank correlation coefficients (ρ) were calculated using R (R Core Team, 2014) to determine correlations between variables, using the suggested critical value of ρ ≥ 0.786 for 5% significance with a sample size of 7 (Zar, 1972).

Principal component and non-metric multidimensional scaling analyses were performed using the vegan package in R (Borcard et al., 2011; Oksanen, 2013). Environmental variables were z-transformed to standardize across different scales and units by subtracting the population mean from each measurement and dividing by the standard deviation. OTU count data were Hellinger-transformed to standardize to relative abundances (Legendre and Legendre, 2012). Other than unweighted UniFrac distances, which were calculated using the online UniFrac portal (Lozupone et al., 2006), distance/dissimilarity indices were calculated using the vegan package in R. All principle component analyses are presented using scaling 1; therefore, the distance between sites on the biplot represents their Euclidean distance, and the right-angle projection of a site onto a descriptor vector shows the approximate position of that site on the vector (Legendre and Legendre, 2012).

## Results and discussion

### Bottom water chemistry

Conductivity generally increased downriver, with upper estuary stations EMP0692 and D4 approximately 100 μS cm^−1^ greater than stations further up either river (Table 1). This change in conductivity was reflected in a marginal correlation with station longitude (ρ = −0.71, *p* = 0.088), as both rivers generally flow westward. Freshwater flows in the Delta are typically high in late winter and spring, followed by a prolonged dry season, during which brackish water from San Francisco Bay gradually moves upriver (eastward; Kimmerer, 2004). During and after periods of high freshwater flow, the conductivity gradient between the rivers to the upper estuary would likely be more pronounced. In contrast, since our samples were collected after months of low precipitation and therefore low Delta outflow (Supplementary Figure A1), it is not surprising that conductivity is only marginally higher in the oligohaline upper estuary than in the rivers.

**Table 1:** **Bottom water chemistry and benthic potential nitrification rates**.

**Station**	**Region**	**Lat**.	**Lon**.	**Conductivity**	**Dissolved oxygen**	**pH**	**Temp**.	**Alkalinity**	**NH^+^_4_**	**NO^−^_3_**	**NO^−^_2_**	**Potential nitrification**
		**°N**	**°W**	**μS cm^−1^**	**mg L^−1^**		**°C**	**mg L^−1^ CaCO_3_**	**μM**	**μM**	**μM**	**nmol NO_X_ g^−1^ h^−1^**
EMP0093	SJ	38.037	121.703	686	6.31	7.7	22.1	119	2.69	14.16	0.43	55.41 (±2.49)
EMP0089	SJ	38.084	121.646	665	5.83	7.6	22.1	110	4.48	16.00	0.72	8.29 (±1.01)
EMP0046	FT	38.018	121.605	704	5.62	8.0	23.1	129	1.08	1.79	0.14	11.38 (±0.66)
EMP0067	Sac	38.139	121.687	675	5.61	7.8	22.1	119	15.43	10.76	0.58	118.74 (±24.86)
SAC01	Sac	38.167	121.597	674	5.62	7.8	22.1	131	27.12	9.71	0.50	146.00 (±70.97)
EMP0692	Est	38.034	121.851	783	7.21	7.6	22.5	120	7.34	22.57	0.62	47.97 (±2.6)
D4	Est	38.058	121.819	775	6.38	7.2	22.4	115	6.55	16.50	0.64	20.33 (±2.2)

*SJ, San Joaquin River; FT, Franks Tract; Sac, Sacramento River; Est, Upper Estuary. Potential nitrification rates were normalized to sediment wet weight; values in parentheses show the standard deviation of triplicate measurements. All data other than nutrient concentrations and potential nitrification rates were provided courtesy of the Southern California Coastal Water Research Project*.

Bottom water nutrient concentrations were generally in the range of values previously reported from northern San Francisco Bay and the Delta (Wankel et al., 2006; Mosier and Francis, 2008; Parker et al., 2012), though with substantial geographical variation (Table 1). NH^+^_4_ was higher in the Sacramento River (stations EMP0067 and SAC01; 15.43 and 27.12 μM, respectively) than in other regions, where NH^+^_4_ ranged from 1.08 to 7.34 μM. NO^−^_3_ was slightly higher in the upper estuary and San Joaquin River stations (14.16–22.57 μM) compared to the rest of the Delta (1.79–10.76 μM). All nutrients were lowest at station EMP0046, located at the southern tip of Franks Tract, a shallow tidal lake connected to the San Joaquin River (Lucas et al., 2002). Compared to the surrounding channels, Franks Tract generally has low turbidity and high productivity (Jassby and Cloern, 2000), which was supported by the relatively high pH (8.0) and low macronutrient concentrations, compared to other sites (Table 1).

### Nitrification

Potential nitrification rates were quantified by measuring the change in NO_X_ concentrations in amended sediment slurries over time. In contrast to measuring rates that more closely approximate *in situ* conditions (i.e., intact core incubations), potential rates estimate the maximal capability of the viable ammonia-oxidizing populations in the slurry, by alleviating any NH^+^_4_ or oxygen limitation. While potential rate measurements are not a perfect proxy for *in situ* processes, rates determined under “optimal” conditions yield insightful information about differences in the maximum sustainable nitrification rate between sites. NO_X_ increased linearly over the incubation period in all Delta samples (*R*^2^ = 0.92–0.99, mean ± SD = 0.98 ± 0.02, *n* = 21). Potential nitrification ranged from 8.3 to 146.0 nmol NO_X_ g^−1^ h^−1^ (58.3 ± 54.2, rates normalized to wet sediment weight; Table 1). Though quite wide, this range was similar to those reported by previous studies using similar methods (Kemp et al., 1990; Joye and Hollibaugh, 1995; Dollhopf et al., 2005; Smith et al., 2014b).

Potential rates at the two Sacramento River stations were an order of magnitude higher than rates from the other study sites (Table 1). This was particularly interesting because of the high concentrations of NH^+^_4_ in the Sacramento River: the correlation between potential nitrification and NH^+^_4_ was positive and at the margins of significance (ρ = 0.71, *p* = 0.088). Due to the recent controversy over the ecological impacts of elevated NH^+^_4_ concentrations in the Sacramento River (Glibert, 2010; Brooks et al., 2012; Cloern et al., 2012; Lancelot et al., 2012), there is substantial interest in quantifying the rates of NH^+^_4_ transformations throughout the Delta. While previous work has inferred high nitrification rates in the Sacramento River due to longitudinal decreases in NH^+^_4_ and increases in NO^−^_3_ concentrations (Parker et al., 2012), no measurements of nitrification rates in the Delta exist to date. Although the potential nitrification rates reported here do not yet allow for an estimate of the *in situ* benthic nitrification rates, they nevertheless suggest that such rates may be higher in the Sacramento River than in the rest of the Delta. Throughout the Delta, there is a clear need to accurately quantify the contribution of nitrification (both benthic and pelagic) to NH^+^_4_ depletion, under conditions that more closely approximate those observed *in situ*. Such an effort would confirm whether the sediments of the Sacramento River are a significant sink for the NH^+^_4_ present in its waters, as our data suggest.

**Figure 2:**
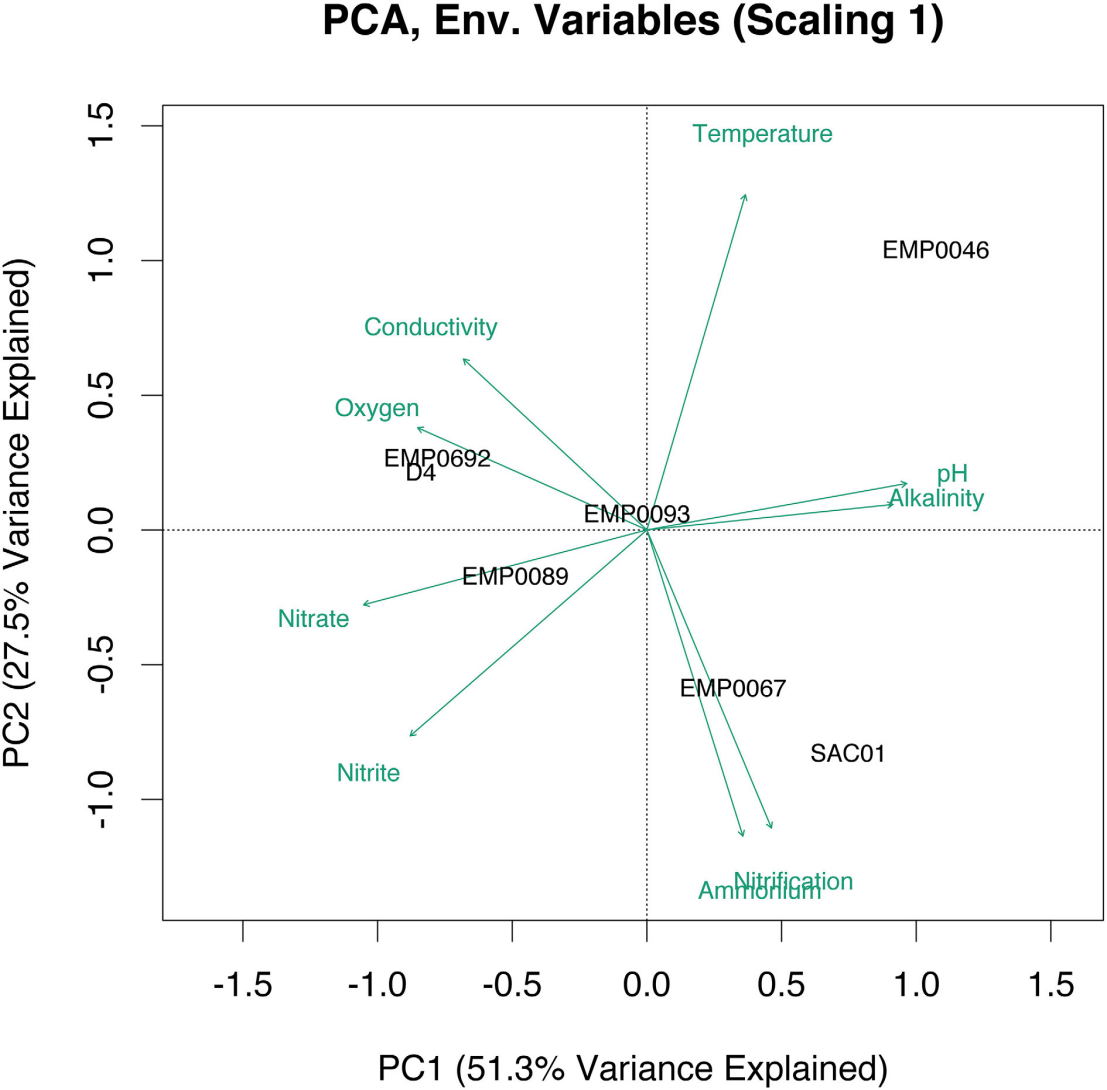
**PCA biplot (scaling 1) of environmental data at the sampled Delta stations**. Data were z-transformed prior to the analysis to standardize across varying units and scales. Distance between stations on the biplot represents Euclidean distance in multidimensional space. Projections of a station onto a green environmental vector indicate its position along that vector.

We used principal component analysis (PCA) to cluster stations according to a combination of environmental variables and nitrification potentials. Combined, the first two eigenvectors explained 78.8% of the variance in bottom water physicochemical parameters, and suggested strong geographical clustering of stations (Figure 2). The upper estuary stations D4 and EMP0692 clustered together, due to higher conductivity, dissolved oxygen, and NO^−^_3_. High NH^+^_4_ and potential nitrification rates separated SAC01 and EMP0067, the two Sacramento River stations, from the rest of the study sites. EMP0046 appeared distinct from all other sites, while EMP0093 and EMP0089 from the San Joaquin River also clustered with one another. Overall, the PCA suggested the four sampled geographical regions of the Delta may be considered distinct physicochemical and biogeochemical regimes.

### Ammonia oxidizer abundances

**Figure 3:**
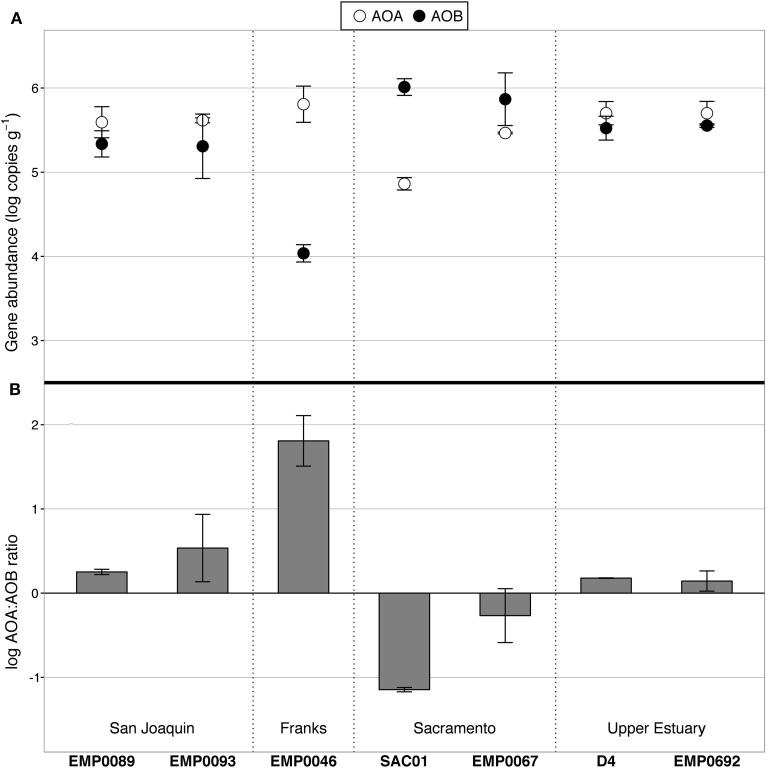
**AOA and AOB *amoA* abundance, as determined by qPCR. (A)** Average values of duplicate samples per site. Abundance data are normalized by wet weight of sediment. Error bars show the range of duplicate samples. AOA are shown in white, AOB in black. **(B)** Log-transformed ratio of AOA:AOB *amoA*. Values above zero indicate AOA abundance greater than AOB, and visa versa. Error bars show the range of values from duplicate extractions per station.

Abundance of AOA and AOB in surface sediments was determined by domain-specific *amoA* qPCR assays. Genes associated with both groups were detectable at all sites, but their abundances varied substantially: AOA *amoA* was present at 7.28 × 10^4^ to 6.42 × 10^5^ copies g^−1^, while AOB *amoA* ranged between 1.09 × 10^4^ and 1.03 × 10^6^ copies g^−1^ (Figure 3; gene copies are normalized to wet sediment weight). As with potential nitrification rates, ammonia oxidizer abundances showed regional patterns: the average log ratio of AOA:AOB *amoA* in the Sacramento River was −0.76, compared to 1.11 in the San Joaquin River and 0.16 in the upper estuary. In other words, AOA outnumbered AOB in the San Joaquin River by 2- to 18-fold, AOB outnumbered AOA in the Sacramento River by 3- to 14-fold, and their abundances were practically equal in the upper reaches of San Francisco Bay. Prior work in the upper estuary also found AOA present at an equal or greater abundance than AOB over multiple summers, though with some site-specific differences in overall abundance values (Mosier and Francis, 2008).

The correlation between AOA abundances and potential nitrification rates was negative but statistically insignificant (ρ = −0.64, *p* = 0.139), while AOB abundances and potential rates were positively correlated at the margins of significance (ρ = 0.71, *p* = 0.088). Although the *in situ* biogeochemical rates and the levels of ammonia oxidizer gene expression are still unknown, these correlations suggest that AOB may have been driving nitrification at those sites in the Delta where benthic nitrification rates were highest. Such a relationship between AOB gene abundance and potential nitrification rates agrees with observations from intertidal sediments in polyhaline Elkhorn Slough, where AOB abundances also strongly correlated with potential rates, while the relationship between AOA abundances and potential rates was much weaker (Smith et al., 2014b). Therefore, AOB appear to be potentially important players in the N cycles of both polyhaline and oligohaline/freshwater estuarine sediments.

Interestingly, the site with the highest bottom water NH^+^_4_ concentration (27.1 μM; station SAC01 in the Sacramento River) also had the highest AOB abundance (1.03 × 10^6^ copies g^−1^). Across all sites, the rank correlation between AOB abundance and bottom water NH^+^_4_ concentration was perfectly positive (ρ = 1, *p* < 0.001). AOB have previously been found to outnumber AOA in the sediments of other eutrophic systems, including Elkhorn Slough (Wankel et al., 2011; Smith et al., 2014b), the organic-rich regions of Lake Taihu (Wu et al., 2010), Fuyang River surface sediments (Wang et al., 2014), and freshwater flow channels fertilized with high doses of NH^+^_4_ (Herrmann et al., 2011). AOB also vastly outnumbered AOA in the extremely NH^+^_4_-rich activated sludge of the Palo Alto wastewater treatment plant (Wells et al., 2009). The numerical dominance of AOB over AOA in NH^+^_4_-rich ecosystems may not be a universal trend, as numerous studies have reported AOA outnumbering AOB in sediments under diverse nutrient regimes (Caffrey et al., 2007; Moin et al., 2009; Abell et al., 2010; Liu et al., 2013); however, the occurrence of such a relationship in numerous ecosystems suggests there is a relationship between benthic AOB abundance and NH^+^_4_ loadings in at least some freshwater and estuarine ecosystems.

### AOA and AOB community diversity

The *amoA* clone libraries in this study are the first exploration of the ammonia-oxidizing communities in the Sacramento-San Joaquin Delta, expanding the database of *amoA* sequences from the San Francisco Bay estuary system (Francis et al., 2005; Mosier and Francis, 2008; Lund et al., 2012) upstream into the Sacramento and San Joaquin rivers. Furthermore, ammonia-oxidizing communities in rivers are undersampled compared to coastal sediments, soils, and marine waters (Biller et al., 2012; Cao et al., 2013), and the data in this study help to expand our understanding of the diversity of *amoA* and ammonia-oxidizing microorganisms in riverine sediments.

**Figure 4:**
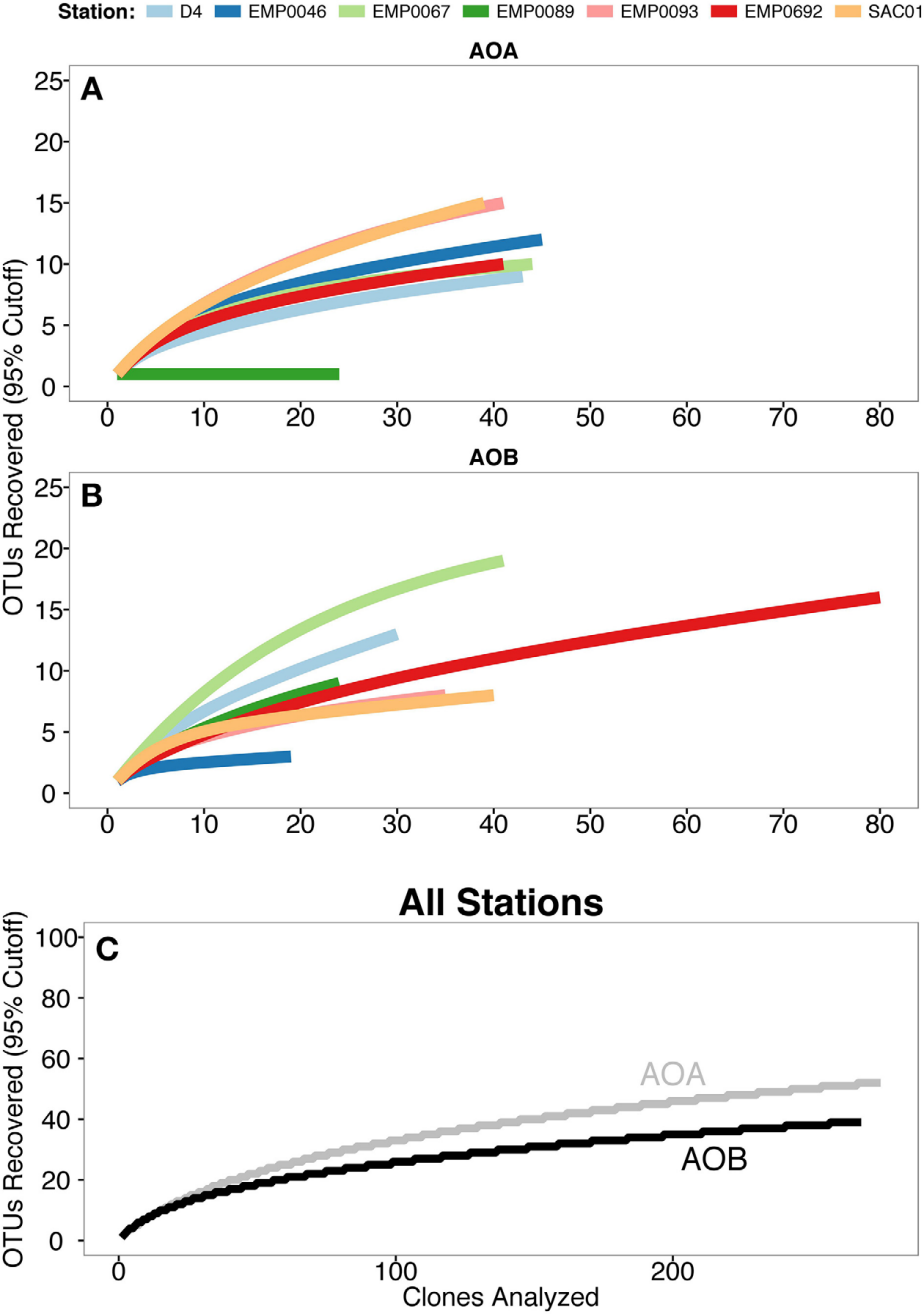
**Rarefaction curves of *amoA* OTU (≥95% similarity) data**. **(A)** AOA and **(B)** AOB, with color denoting sampling site. **(C)** Combined OTU data from all stations, with AOA *amoA* data in gray and AOB *amoA* in black.

Both archaeal and bacterial *amoA* sequences were obtained from all 7 Delta sites. The richness of AOA *amoA* was greater than for AOB: when analyzed using a 95% sequence similarity cutoff, the 276 total AOA *amoA* sequences formed 52 OTUs, while the 269 AOB *amoA* sequences formed 39 OTUs (Table 2). Rarefaction analysis of AOA OTUs suggested the highest richness at stations SAC01 in the upper Sacramento River and EMP0093 in the lower San Joaquin (Figure 4A), while richness was remarkably low at EMP0089, further up the San Joaquin River. It is unclear why this station showed such a marked lack of AOA diversity, especially since AOB *amoA* sequences obtained here were far more diverse (see below). Rarefaction curves of AOB OTUs differed from those of AOA: stations EMP0067 and D4 had the highest richness, while EMP0046 had the lowest (Figure 4B). Curves generated with all combined sequences appeared to be leveling off but not yet at a plateau, indicating that we characterized the majority, but not the fullest extent, of the total ammonia oxidizer diversity in the Delta (Figure 4C), and that many OTUs present were shared between sites.

**Table 2:** **Diversity (Chao1 index) and richness (Shannon index) data for Delta *amoA* OTU data**.

	**No. Clones**	**No. OTUs**	**Unique OTUs**	**Chao1**	**Shannon**
**AOA**					
All Sites	276	52		76 (69.7%)	3.1
EMP0093	41	15	6	16 (93.8%)	2.4
EMP0089	24	1	0	1 (100%)	0
EMP0046	45	12	9	15 (80.0%)	2.1
EMP0067	44	10	2	12 (83.3%)	1.9
SAC01	39	15	10	24 (62.5%)	2.3
EMP0692	41	10	4	12 (83.3%)	1.9
D4	43	9	3	12 (75.0%)	1.6
**AOB**					
All Sites	269	39		52 (75.0%)	2.9
EMP0093	35	8	0	9 (88.9%)	1.6
EMP0089	24	9	3	12 (75.0%)	1.7
EMP0046	19	3	1	3 (100%)	0.8
EMP0067	41	19	8	22 (86.4%)	2.8
SAC01	40	8	0	11 (72.7%)	1.8
EMP0692	80	16	7	34 (47.1%)	1.8
D4	30	13	2	27 (48.1%)	2.3

*Parenthetical numbers next to the Chao1 indices show the percent of estimated (Chao1) diversity actually recovered (number of OTUs). “Unique” OTUs for each station show the number of OTUs exclusively containing sequences from that station. OTUs were defined as ≥95% sequence similarity*.

To further assess the richness and diversity of the Delta sediments, Chao1 and Shannon indices were calculated for individual sites (Table 2). The Chao1 richness index, which estimates the total theoretical number of OTUs in a sample (Chao, 1984), ranged from 1 to 24 for AOA (mean ± SD = 13 ± 7 OTUs), and indicated that Sacramento River station SAC01 harbored the highest AOA richness. Station SAC01 also had the highest number (10) of unique AOA OTUs. Chao1 suggested 3 to 34 AOB OTUs should be present at Delta sites (17 ± 11 OTUs), with the two upper estuary stations having the greatest estimated richness. The fraction of estimated OTUs observed in the combined data sets was 69.7% for AOB and 75% for AOA, supporting the conclusion that a substantial number of OTUs from the Delta may yet be unrecovered (Figure 4C). The percentage of richness recovered at each individual station ranged from 62.5 to 100% (83.0 ± 13.3%) for AOA and 47.1–100% (74.2 ± 18.7%) for AOB.

The Shannon diversity index estimates the “entropy” associated with OTU composition in a data set, or the uncertainty of predicting the identity of a randomly selected OTU (Legendre and Legendre, 2012). While richness indices estimate the total number of OTUs in a sample, diversity indices also account for the relative abundance (“evenness”) of OTUs within sites. Shannon analysis generally supported the conclusions of the Chao1 index, though with a few interesting differences. While the Sacramento River station SAC01 had the highest AOA richness as estimated by the Chao1 index, the Shannon index matched the AOA rarefaction analyses in suggesting that the San Joaquin River station EMP0093 (Shannon = 2.4), as well as SAC01 (Shannon = 2.3), had relatively high diversity (Table 2; Figure 4A). For AOB data, Shannon indices indicated *amoA* diversity at Sacramento River station EMP0067 and upper estuary station D4 was high (Shannon = 2.8 and 2.3, respectively; Table 2), matching the AOB rarefaction curve (Figure 4B). Both Chao1 and Shannon indices were in the general range of other estuarine and riverine sediments, though on the higher side (e.g., Mosier and Francis, 2008; Liu et al., 2013; Zheng et al., 2014). Neither Shannon nor Chao1 indices were significantly correlated with potential nitrification rates.

### AOA *amoA* composition

Years after the discovery of “Marine Group I” Crenarchaeota in the ocean (DeLong, 1992; Fuhrman et al., 1992), these archaea have been broadly (though not exclusively) split into “group I.1a” (marine water and sediment) and “group I.1b” (soil) clades, and appear to be abundant members of the microbial communities in soils, sediments, freshwaters, and marine waters. Numerous cultures and enrichments have documented their ability to sustain autotrophic growth by oxidizing ammonia (Könneke et al., 2005; Santoro and Casciotti, 2011; Tourna et al., 2011; French et al., 2012; Mosier et al., 2012b). In initial studies of AOA *amoA* in San Francisco Bay, many sequences from freshwater and oligohaline sediments grouped together in a distinct and presumably “low-salinity” I.1a clade (Francis et al., 2005; Mosier and Francis, 2008); sequences in this clade are commonly found in diverse freshwater and oligohaline estuarine environments (Mosier and Francis, 2008; Hu et al., 2010; Wu et al., 2010; Wankel et al., 2011; Liu et al., 2013; Sonthiphand et al., 2013). A number of putative low-salinity AOA have been enriched from environmental samples, including *Nitrosoarchaeum limnia* strain SFB1 (Blainey et al., 2011; Mosier et al., 2012b), *N. limnia* strain BG20 (Mosier et al., 2012a), *N. koreensis* (Jung et al., 2011), and freshwater enrichment AC2 (French et al., 2012).

**Figure 5:**
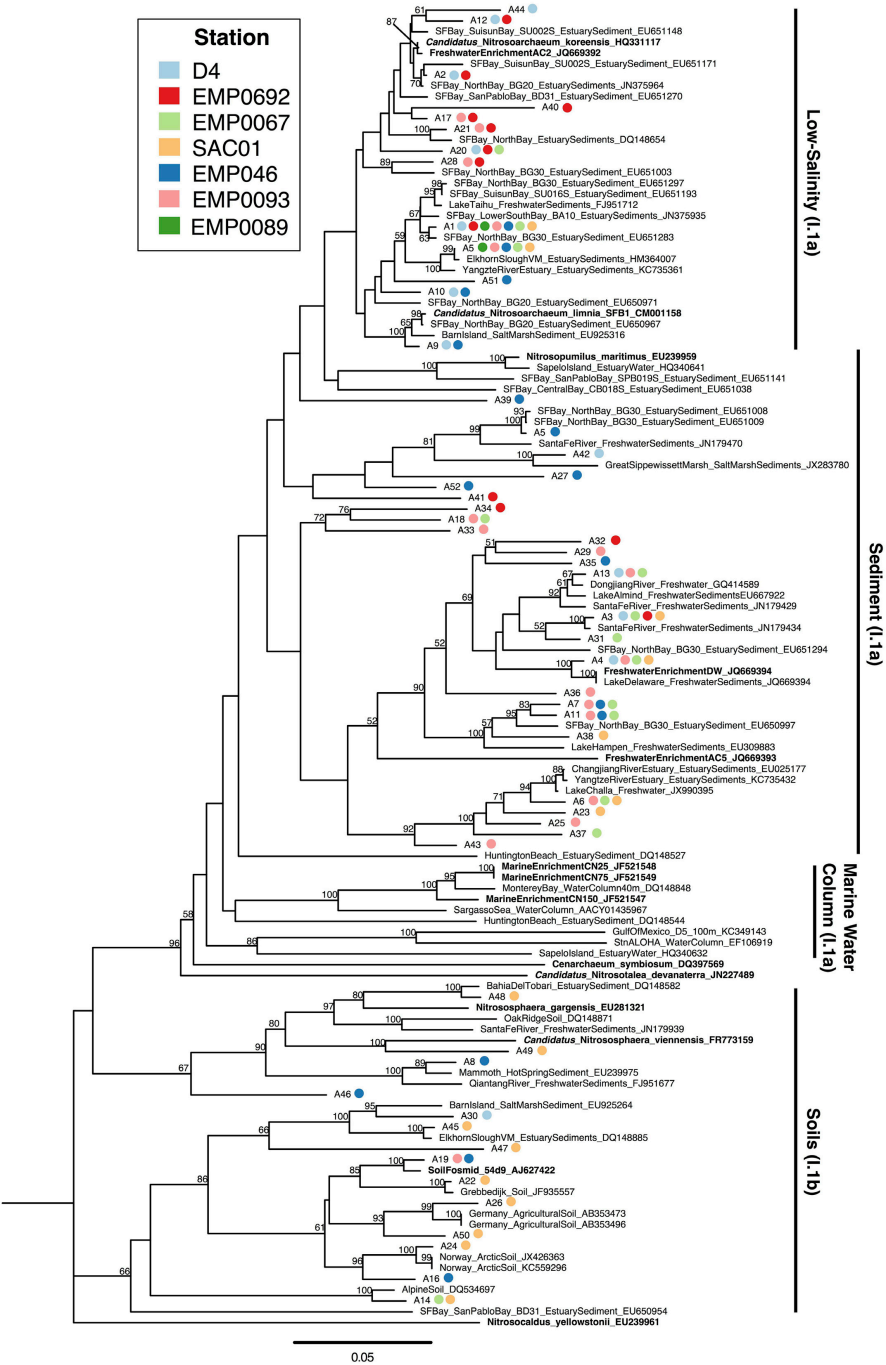
**Neighbor joining tree of AOA *amoA* OTUs (≥95% similarity) from the Delta**. Colored points next to each OTU indicate the composition of the OTU by station. Values at nodes indicate bootstrap support ≥50% (1000 neighbor-joining bootstrap replicates). Reference sequences from named organisms are indicated in bold. Clades referred to in the text are marked to the right of the tree.

In our clone libraries, 25% (13/52) of AOA OTUs fell within the “low-salinity” clade (Figure 5). The only AOA OTU that contained sequences from all 7 Delta sites (“A1”) clustered within this clade, near another OTU (“A5”) containing sequences from all 5 riverine stations. Both of these ubiquitous OTUs were similar to *N. limnia* (94.9–95.3% nucleotide similarity to the *N. limnia* reference *amoA* sequence). OTU “A1” was the most populous Delta OTU, containing 86/276 (31%) of Delta AOA sequences, and the combined OTUs within the “low-salinity” clade included 152/276 (55%) of the Delta AOA sequences. Numerous OTUs (8/52; 15.4%), all of which contained sequences from the upper estuary stations, grouped near *N. koreensis* and freshwater enrichment AC2, suggesting this clade may be well suited to live in oligohaline sediments.

Nearly half the AOA OTUs obtained from the Delta (25/52; 48%) grouped with I.1a AOA sequences from marine, estuarine, and freshwater sediments (related to *Nitrosopumilus maritimus*) to form three major “sediment” clades (Figure 5). No Delta OTUs clustered closely with *N. maritimus*, despite previous detection of *N. maritimus*-like sequences in San Francisco Bay sediments (Mosier and Francis, 2008) and other estuaries (Cao et al., 2013), which suggests this clade of AOA does not penetrate inland to the rivers upstream of San Francisco Bay. Within the sediment clades, 11 OTUs (21% total) clustered in a clade (90% bootstrap support) containing freshwater enrichments “AC5” and “DW” (both isolated from lakes in Ohio, USA). Physiological experiments showed these strains grow well at relatively low NH^+^_4_ concentrations and are not impaired by low or fluctuating oxygen concentrations, perhaps as a specialization for living at the sedimentary oxic/anoxic interface (French et al., 2012). These organisms appeared to be consistent members of the benthic AOA communities throughout all stations in the Delta. Notably, OTU “A4,” containing sequences from both rivers and the upper estuary, clustered tightly with enrichment “DW” (100% bootstrap support and 98.4% nucleotide similarity). This AOA strain therefore appears to be common in the Delta, and may be a generally important player in N cycling in freshwater and oligohaline sediments.

AOA group I.1b, typically recovered from soils (Gubry-Rangin et al., 2011; Pester et al., 2012), was also well represented in our libraries. In total, 27% (14/52) of OTUs from the Delta were related to two distinct I.1b clades: one contained *Nitrososphaera* spp. (Hatzenpichler et al., 2008; Tourna et al., 2011), while the other included soil fosmid 54d9, the first metagenomic linkage between an archaeal *amoA* gene and a 16S rRNA gene (Figure 5; Treusch et al., 2005). A small number of OTUs (8%, 4/52), comprising only sequences obtained from Sacramento River station SAC01 or Franks Tract station EMP0046, grouped with the *Nitrososphaera* sequences. Interestingly, these stations appear to harbor common AOA lineages despite the markedly different physicochemical conditions between them (Table 1). On the other hand, the clade containing fosmid 54d9 contained 10/52 Delta OTUs (19%), and was geographically widespread, including sequences from all regions in the Delta. It is possible some uncultured group I.1b AOA have simply been transported into the Delta from soils in its agricultural watershed, as much of the Delta itself is active farmland (Lund et al., 2010) and group I.1b AOA are commonly found in agricultural soils (Nicol et al., 2008; Pratscher et al., 2011); however, without sequencing *amoA* mRNA transcripts, we could not determine whether their presence within clone libraries was not due to inactive passage through the Delta. Nevertheless, the presence of I.1b AOA in many other estuarine and riverine sediments (Mosier and Francis, 2008; Moin et al., 2009; Abell et al., 2010; Wankel et al., 2011; Liu et al., 2013) implies that they are at least a common member of ammonia-oxidizing communities in these systems.

Sequences clustering with *Nitrosotalea devanaterra* have been found in numerous freshwater environments, including lakes and rivers, and are occasionally found in estuary sediments (Cao et al., 2013). The lack of this clade in our AOA *amoA* clone libraries is curious. As *N. devanaterra* was originally isolated from, and commonly found in, acidic soils (Gubry-Rangin et al., 2011; Lehtovirta-Morley et al., 2011), these AOA may be poorly adapted to the pH-neutral regions of the Delta (Table 1). However, while a negative correlation between *Nitrosotalea* relative abundance and pH was documented from lakes in the Pyrenees (Auguet and Casamayor, 2013), *Nitrosotalea* sequences have also been reported from numerous non-acidic freshwater ecosystems (Auguet et al., 2012; Liu et al., 2013; Bollmann et al., 2014). It is unclear what factors other than pH may regulate the abundance or activity of *Nitrosotalea* AOA in freshwater ecosystems. If the lack of this clade in the Delta is not merely an artifact of our limited number of sequenced clones, comparing the environmental conditions of the Delta with sediments with abundant *Nitrosotalea* may be useful for identifying factors that potentially affect its distribution.

Overall, our data showed a diverse AOA community in the Delta, including many of the clades commonly found in estuary and freshwater sediments. Unlike other ecosystems where AOA *amoA* is restricted to a small number of clades (e.g., the marine water column and terrestrial soils), AOA diversity in estuary sediments is often quite complex, typically including sequences from marine, low-salinity, and soil clades (Biller et al., 2012; Cao et al., 2013). Previous work from a range of sites in San Francisco Bay found the majority of AOA sequences clustering in either the “low-salinity” clade (now including *Nitrosoarchaeum* spp.), one of the major soil clades, and a diverse sediment clade including *Nitrosopumilus maritimus*, though numerous additional sequences fell in other less populous clades (Francis et al., 2005; Mosier and Francis, 2008). Similarly diverse benthic archaeal communities have been reported from other estuaries, including Plum Island Sound (Bernhard et al., 2010), the Westerschelde estuary (Sahan and Muyzer, 2008), Elkhorn Slough (Francis et al., 2005; Wankel et al., 2011; Smith et al., 2014b), and the Changjiang Estuary (Dang et al., 2008), among others. However, the geographic positioning of the Delta at the upstream reaches of San Francisco Bay was reflected in AOA communities largely dominated by OTUs grouping in the soil and freshwater clades, with fewer OTUs grouping in typical marine clades. For example, the preponderance of Delta sequences related to *Nitrosoarchaeum* spp., as well as sequences grouping with freshwater enrichments “DW” and “AC5,” indicated the widespread presence of these strains in this upper estuary and riverine ecosystem, while the absence of OTUs closely related to *Nitrosopumilus maritimus* suggested this organism is less common in freshwater sediments than in estuarine or marine sediments. Compared to estuary and marine sediments, AOA community composition in freshwater (and especially river) sediments is relatively understudied (Cao et al., 2013). It is therefore difficult to assess the “typical” diversity of freshwater sediments. However, a handful of studies have documented diverse benthic freshwater AOA communities, commonly including the soil, “low-salinity,” and some marine/estuary sediment clades (Herrmann et al., 2009; Wu et al., 2010; Liu et al., 2013; Bollmann et al., 2014), suggesting the clades of AOA recovered from the riverine Delta stations may include common members of benthic freshwater AOA communities. Compared to marine waters and sediments, the presence of group I.1b *amoA* OTUs in the Delta may reflect the proximity of this river-dominated system to terrestrial influences.

### AOB *amoA* composition

**Figure 6:**
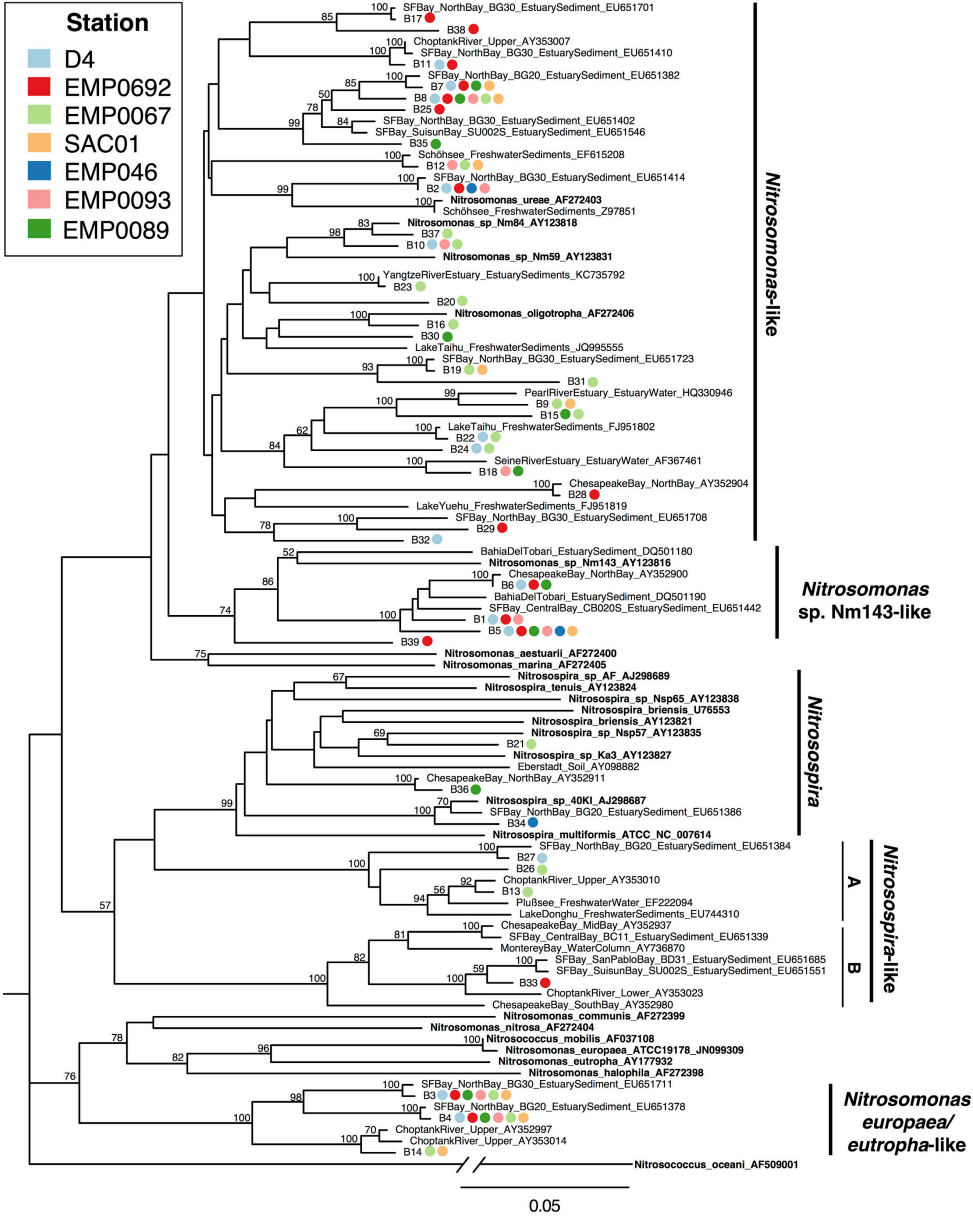
**Neighbor joining tree of AOB *amoA* OTUs (≥95% similarity) from the Delta**. Colored points next to each OTU indicate the composition of the OTU by station. Values at nodes indicate bootstrap support ≥50% (1000 neighbor-joining bootstrap replicates). Reference sequences from named organisms are indicated in bold. Clades referred to in the text are marked to the right of the tree.

In estuary sediments, AOB often show distinct phylogenetic partitioning along salinity gradients (e.g., Francis et al., 2003; Bernhard et al., 2005), with communities in marine-influenced regions generally dominated by *Nitrosospira*-like *amoA* sequences and those in oligohaline/freshwater regions by a mix of *Nitrosospira*-like and *Nitrosomonas*-like sequences (Bernhard and Bollmann, 2010). These two major AOB clades appear physiologically distinct. For example, both lab and field experiments have suggested *Nitrosospira*-like AOB can be more active or abundant in low NH^+^_4_ environments, whereas *Nitrosomonas*-like AOB may be adapted to grow in high NH^+^_4_ environments (Taylor and Bottomley, 2006; Peng et al., 2013), although modeling experiments have suggested cultured *Nitrosospira* may grow rapidly in response to large NH^+^_4_ pulses (Bouskill et al., 2012b). *Nitrosospira*-like AOB are common in marine and soil ecosystems (Stephen et al., 1996; Rotthauwe et al., 1997; Freitag and Prosser, 2003; O'Mullan and Ward, 2005), and often found in estuaries, as well (Francis et al., 2003; Bernhard et al., 2005; Freitag et al., 2006; Mosier and Francis, 2008; Smith et al., 2014b). However, only 7/39 (17.9%) Delta OTUs grouped with *Nitrosospira* or *Nitrosospira*-like sequences. Along with sequences previously recovered from soils and estuary sediments, 3/39 OTUs (7.7%) clustered near cultured *Nitrosospira* sequences, including *N. multiformis* and *N. briensis*. OTU “B34” was very closely related to *Nitrosospira*. sp. 40K1 (96.9% nucleotide identity), originally isolated from loam soil (Jiang and Bakken, 1999). Only one OTU (“B33”) was within the mesohaline *Nitrosospira*-like clade (“B”) common in estuaries, while 3/39 OTUs (7.7%) grouped in the low-salinity *Nitrosospira*-like clade (“A”) (Figure 6). Overall, *Nitrosospira*-like OTUs contained sequences from 5 of 7 sites, and were therefore relatively widespread throughout the Delta. However, no OTUs in this clade contained sequences from more than one station, suggesting the *Nitrosospira*-like AOB in the Delta may be sensitive to site-specific environmental conditions.

Three AOB OTUs fell in a well-supported clade that grouped, with moderate support, next to a cluster of *Nitrosomonas* species (*Nitrosomonas europaea*, *N. eutropha*, *N. communis, N. nitrosa, N. halophila*, etc.) generally adapted to high-N environments (Koops et al., 1991; Stehr et al., 1995; Mortimer et al., 2004; Stein et al., 2007; Dang et al., 2010). This as yet-uncultivated clade, referred to here as “*Nitrosomonas europaea/eutropha*-like,” has been found in a range of nutrient-rich ecosystems (Sahan and Muyzer, 2008; Wankel et al., 2011; Zheng et al., 2014). OTUs “B3” and “B4” contained sequences from all Delta sites other than Franks Tract, and were nearly identical (>99% nucleotide identity) to sequences within this clade previously obtained from northern San Francisco Bay (Mosier and Francis, 2008). Notably, all three *Nitrosomonas europaea/eutropha*-like OTUs included sequences from both NH^+^_4_-rich Sacramento River stations (Figure 6). Closely related sequences have also been obtained from low-salinity, high-nutrient sediments in the upper Choptank River, Maryland, though NH^+^_4_ in the Choptank was lower than the Sacramento River (Francis et al., 2003). These OTUs appear to represent organisms broadly adapted to oligohaline and relatively N-rich environments, and the absence of sequences from Franks Tract suggests its relatively oligotrophic (see Table 1, Figure 2) conditions may not be hospitable to *N. europaea/eutropha-like AOB*.

The majority of AOB OTUs from the Delta (29/39; 74%) were in a broad group referred to here as “*Nitrosomonas*-like” (Figure 6), closely related to cultivated strains including *N. oligotropha* and *N. ureae*. Nucleotide identity between Delta OTUs and sequences from *Nitrosomonas* spp. was 81.3 to 98.0%. *Nitrosomonas ureae* and *N. oligotropha* have relatively high affinity for NH^+^_4_ (Koops and Pommerening-Röser, 2001; Bollmann et al., 2002); thus, these bacteria may be able to thrive in low-NH^+^_4_ environments. Related sequences are commonly found in freshwater and estuarine sediments (Speksnijder et al., 1998; de Bie et al., 2001; Cébron et al., 2003; Bernhard et al., 2005; Peng et al., 2013), including the northern reaches of San Francisco Bay (Mosier and Francis, 2008), and have also been recovered from marine environments (Ward et al., 2007). Some *Nitrosomonas*-like OTUs, such as OTU “B8,” appeared widely distributed across the Delta. However, others were region-specific: OTUs “B11,” “B17,” and “B38” exclusively contained sequences from the upper estuary, and OTU “B2” contained sequences from all regions other than the Sacramento River (Figure 6). *Nitrosomonas*-like OTUs therefore appeared to be common in Delta sediments, but with geographical distributions differing between OTUs.

Multiple AOB OTUs grouped in a clade (86% bootstrap support) containing *Nitrosomonas* sp. Nm143, a strongly-supported, novel AOB lineage (Purkhold et al., 2003) commonly found in marine and estuarine sediments (e.g., Bernhard et al., 2005; Mosier and Francis, 2008). Nucleotide identity between these OTUs and *Nitrosomonas* sp. Nm143 was 86.4–88.0%. All OTUs in this group contained sequences from one or both upper estuary stations, and two contained sequences from both estuarine stations and a San Joaquin River station (Figure 6). OTU “B5” was widely distributed throughout the Delta, containing sequences from all Delta sites other than station EMP0067 (although sequences from SAC01, further up the Sacramento River, were present). These data suggested *Nitrosomonas* sp. Nm143-like AOB were common members of microbial communities of the Delta.

Similar to AOA diversity in the Delta, its diverse AOB communities included many common estuary and freshwater clades, but were distinct from typical benthic meso/polyhaline estuary communities. In estuaries, common AOB clades include those related to *Nitrosomonas ureae/oligotropha/aestuarii*, as well as yet-uncultivated clades close to *Nitrosomonas* sp. Nm143 and *Nitrosospira* (Francis et al., 2003; Bernhard et al., 2005; Mosier and Francis, 2008; Bernhard and Bollmann, 2010; Peng et al., 2013). In freshwater sediments, trophic status and nutrient loading are frequently thought to affect AOB diversity, with *Nitrosomonas europaea/eutropha*-like AOB typically found in eutrophic sediments, *Nitrosospira*-like AOB in lower nutrient sediments, and *Nitrosomonas oligotropha/ureae* generally widespread (Zhang et al., 2007; Chen et al., 2009; Herrmann et al., 2009; Wu et al., 2010; Bollmann et al., 2014). While some common estuary AOB sequences were found in the Delta, few Delta sequences grouped in the *Nitrosospira*-like clade. A number of OTUs, however, grouped with *Nitrosomonas* sp. Nm143-like AOB. All three OTUs in this clade included sequences from both upper estuary stations, as well as at least one river, suggesting the AOB represented by these OTUs are widespread in the upper estuary but also sometimes present in the fresher regions of the Delta, as well. Interestingly, sequences from all regions of the Delta fell in the *Nitrosomonas europaea/eutropha*-like clade, despite the clear physicochemical and trophic differences between the two rivers (Table 1; Figure 2), and a number of Delta OTUs similar to *N. oligotropha/ureae* (though not all) were also widely distributed throughout the Delta. These ubiquitous OTUs, typical of either eutrophic or oligotrophic freshwater sediments, suggest AOB communities in the Delta may be structured by more than just trophic status.

### Geographical clustering of ammonia-oxidizing populations

**Figure 7:**
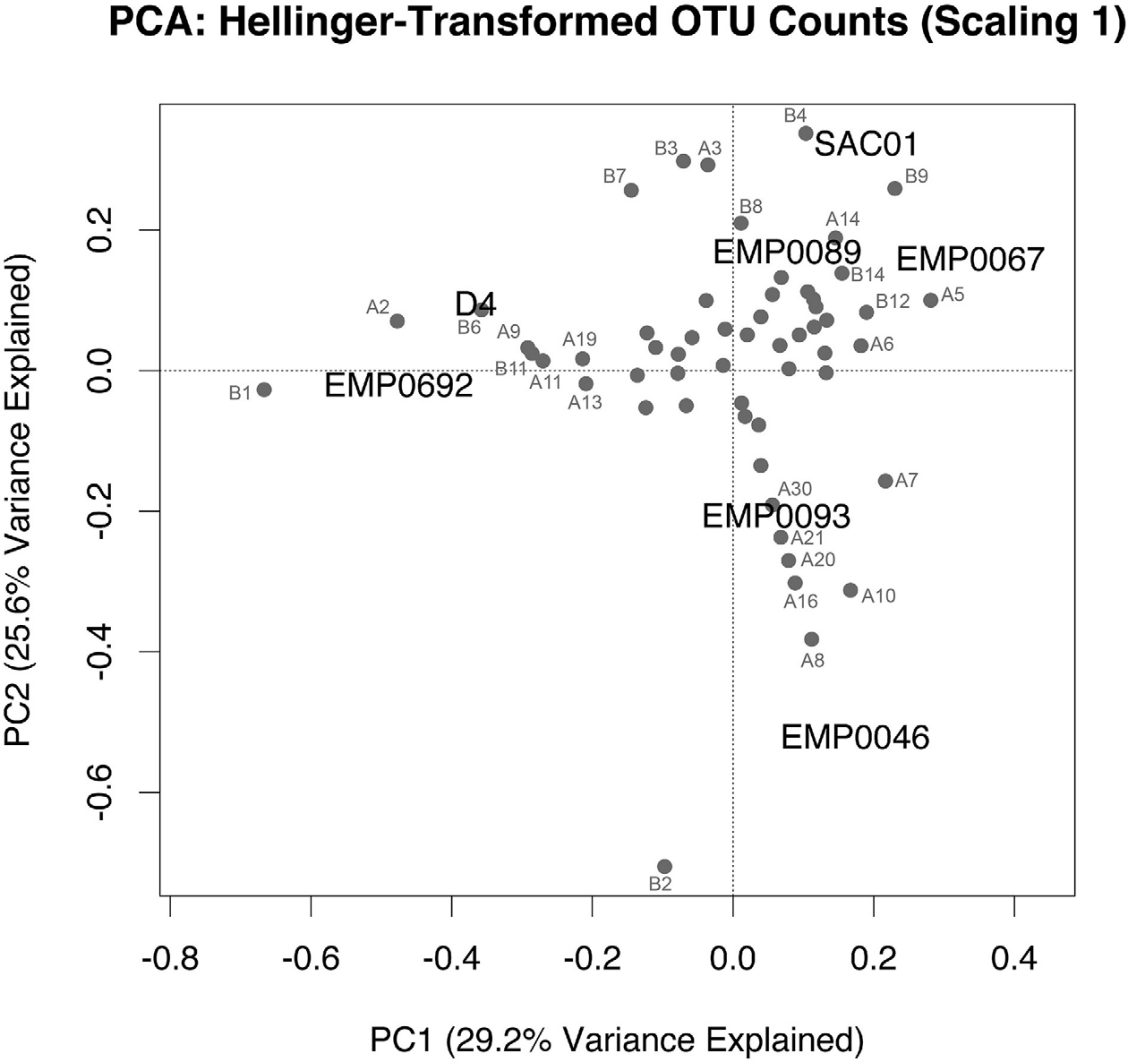
**PCA biplot (scaling 1) of combined, Hellinger-transformed AOA and AOB *amoA* OTU counts**. Distance between stations on the biplot represents Euclidean distance in multidimensional space. Projections of a station onto an OTU vector indicate its position along that vector. Identity of OTUs is indicated, using the same names as Figures 5, 6 (AOA *amoA* OTUs are labeled “A” and AOB *amoA* OTUs are labeled “B”). OTUs clustered near the center are unnamed, for legibility.

To assess whether ammonia oxidizer diversity in the Delta differed by region, we performed PCA on Hellinger-transformed OTU counts of combined AOA and AOB data from each site (analyses of each separate gene showed similar results; Supplementary Figure A2). The Hellinger transformation maintains information on relative abundance between sites, while removing potential effects of differences in total abundance (Legendre and Gallagher, 2001; Legendre and Legendre, 2012). Since clone library data is considered semi-quantitative at best (PCR and cloning biases may cause some clades to be over-represented), we believed transformed OTU counts were a reasonable, if imperfect, representation of relative abundance (a PCA using OTU presence/absence produced similar results; Supplementary Figure A3). The first two principal component axes of the PCA explained 54.8% of the variance in *amoA* diversity between stations. Station EMP0046 grouped alone, due to the effects of numerous archaeal and bacterial OTUs (Figure 7). The distinct ammonia-oxidizing community here may reflect the environmental uniqueness (i.e., low nutrient concentrations and elevated pH) of Franks Tract compared to the other sampled regions (Table 1; Figure 2). The upper estuary stations EMP0692 and D4 clustered together, driven by the presence of multiple OTUs, most notably “B1” and “A2.” Therefore, ammonia oxidizer community composition shifted between the rivers and the upper reaches of San Francisco Bay: even though stations D4 and EMP0692 were only in the low-salinity estuary regime, there is a clear difference between these sites and those in the fresher regions of the Delta. Previous research showed stark differences between ammonia-oxidizing communities along the entire salinity gradient of San Francisco Bay (Mosier and Francis, 2008), and our data suggest that communities in the oligohaline upper estuary were also distinct from those in river sediments. Such partitioning fits the general pattern of distinct ammonia-oxidizing communities along salt gradients (Bernhard and Bollmann, 2010). Additionally, this finding is important in light of recent research suggesting a landward increase in salinity throughout San Francisco Bay and the Delta, due to expected sea level rise and altered precipitation/snowmelt brought on by climate change (Cloern et al., 2011). Understanding the distribution of biogeochemically-relevant microbes throughout this system, and ultimately the controls on their distribution and activity, are crucial aspects of understanding how the ecosystem will respond to environmental changes.

Because the Sacramento River sites had high NH^+^_4_ concentrations and high potential nitrification rates (Table 1), we were particularly interested in whether the ammonia-oxidizing communities in these sediments were distinct from other regions. However, this was not entirely the case: the San Joaquin station EMP0089 grouped near both Sacramento stations (Figure 7), though the Sacramento River stations were more strongly separated when OTU presence/absence data were used (Supplementary Figure A3). Therefore, ammonia oxidizer community composition may not be a strong indicator of biogeochemical potential in sediments in the Delta. However, given the high AOB abundance in the Sacramento River (Figure 3), it is likely that OTUs “B4” and “B9” are particularly important ammonia oxidizers here, as these two bacterial OTUs contributed to the clustering of these three stations in the combined PCA. These OTUs are quite phylogenetically distinct (only 74.7% nucleotide identity): one grouped in the “*Nitrosomonas europaea*/*eutropha*-like” clade, and the other in an unresolved but distinct “*Nitrosomonas*-like” clade (Figure 6).

**Figure 8:**
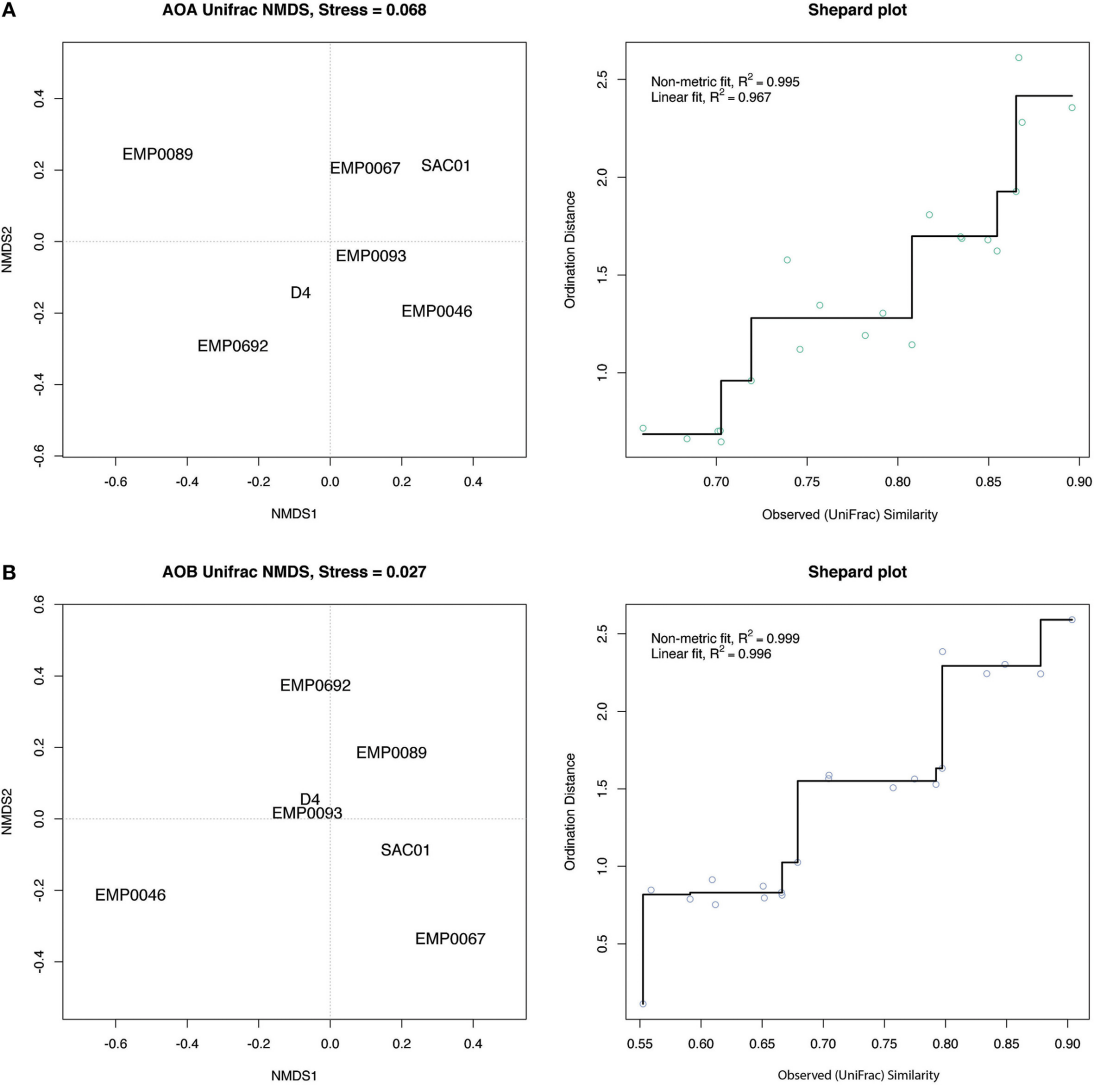
**Unweighted UniFrac distance-based NMDS plots**. **(A)** AOA and **(B)** AOB *amoA*. Proximity between stations represents similarity but is not necessarily equal to original distances. For each gene, the NMDS plot is shown on the left, and a Shepard plot, showing the correlation between observed similarity (i.e., UniFrac distance) and ordination distance, is shown on the right. Each point corresponds to pairwise distances between two sites, and the fit between the UniFrac distances and ordination distances is drawn as a line. Values of R^2^ are shown for a linear correlation and a non-metric fit based on the stress statistic (see Oksanen, 2013).

In addition to the PCA using OTU count data, we analyzed the phylogenetic uniqueness of the Delta stations using non-metric multidimensional scaling (NMDS) of unweighted UniFrac distances (Lozupone and Knight, 2005). While analyses of OTU counts separate sites due to the presence and/or relative abundance of OTUs, UniFrac estimates the fraction of tree branch lengths unique to each site. NMDS compares calculated pairwise distances between sites (in this case, UniFrac distances) to distances between points in ordination space, rearranging the points to maximize the correlation between these two distances and thus minimize a “stress” statistic. Therefore, clustering of sites indicates similarity, but ordination distances do not necessarily reflect the original pairwise distances (Ramette, 2007; Legendre and Legendre, 2012). In contrast to the OTU-count PCA, both AOA and AOB UniFrac NMDS analyses grouped the Sacramento River stations SAC01 and EMP067 separately from the other stations (Figure 8). Thus, even if relative OTU abundance did not clearly separate these stations from other regions, it appears the Sacramento River sediments harbored phylogenetically distinct ammonia-oxidizing populations. In light of their high NH^+^_4_ exposure and potential nitrification rates, the separation of the Sacramento River stations in UniFrac analyses for both genes, combined with the distinct prevalence of large AOB populations, suggested the unique diversity of ammonia oxidizers here may be responsible for the enhanced potential biogeochemical functioning in this river. The upper estuary stations, however, did not group as distinctly in NMDS analyses as in the OTU-count PCA: while the AOA NMDS grouped D4 and EMP0692 somewhat close together, the AOB NMDS did not, grouping them instead with the San Joaquin River stations (Figure 8). While the relative abundances of ammonia-oxidizing OTUs in the upper estuary separated upper estuary stations from the other regions of the Delta (Figure 7), the OTUs driving this separation may not have been phylogenetically unique to this region.

## Conclusions

This study of the Sacramento/San Joaquin Delta indicated benthic ammonia oxidizers are likely important players in the N cycle within this ecosystem, particularly in the NH^+^_4_-replete Sacramento River. The high abundance of AOB in this river (outnumbering AOA), combined with elevated potential nitrification rates, suggested AOB may be driving nitrification in the benthic regions of the Delta most directly affected by anthropogenic NH^+^_4_ pollution. Our clone libraries vastly expanded the database of *amoA* sequences from the San Francisco Bay ecosystem, as well as from freshwater and riverine sediments in general. Across the regions of the Delta, relative abundance of ammonia oxidizer OTUs showed a distinction between the oligohaline estuary and the riverine regions, without clear partitioning of the two rivers. However, there was a marked difference in phylogenetic uniqueness for both AOA and AOB between the Sacramento River and the other regions. Benthic nitrification is likely a key link in the N cycle of estuarine and riverine ecosystems that are strongly affected by anthropogenic nutrient inputs, including the Delta. Understanding the controls on biogeochemical cycles and the associated microbial communities, as well as the relationships between the two, is vital for understanding how aquatic ecosystems will be affected by the climatic changes and increased human development associated with the twenty-first century.

### Conflict of interest statement

The authors declare that the research was conducted in the absence of any commercial or financial relationships that could be construed as a potential conflict of interest.
