# Supplementary material for: Benthic ammonia oxidizers differ in community structure and biogeochemical potential across a riverine delta
Source: Front Microbiol. 2015 Jan 8;5:743. doi: 10.3389/fmicb.2014.00743 (PMC4287051; doi:10.3389/fmicb.2014.00743)
Supplement: Supplementary file 1 [file DataSheet1.DOCX]

***Supplementary Material***

**Benthic Ammonia Oxidizers Differ in Community Structure and Biogeochemical Potential Across a Riverine Delta**

**Julian Damashek^1^, Jason M. Smith^1†^, Annika C. Mosier^1††^, Christopher A. Francis^1^***

^1^Stanford University, Department of Environmental Earth System Science, Stanford, CA, USA

**^†^**Current address: Monterey Bay Aquarium Research Institute, Moss Landing, CA, USA.

**^††^**Current address: University of Colorado Denver, Department of Integrative Biology, Denver, CO, USA.

*** Correspondence:** Christopher A. Francis, Stanford University, Department of Environmental Earth System Science, 473 Via Ortega Room 140, Stanford, CA, 94305-4216, USA.

caf@stanford.edu

1. **Supplementary Figures**


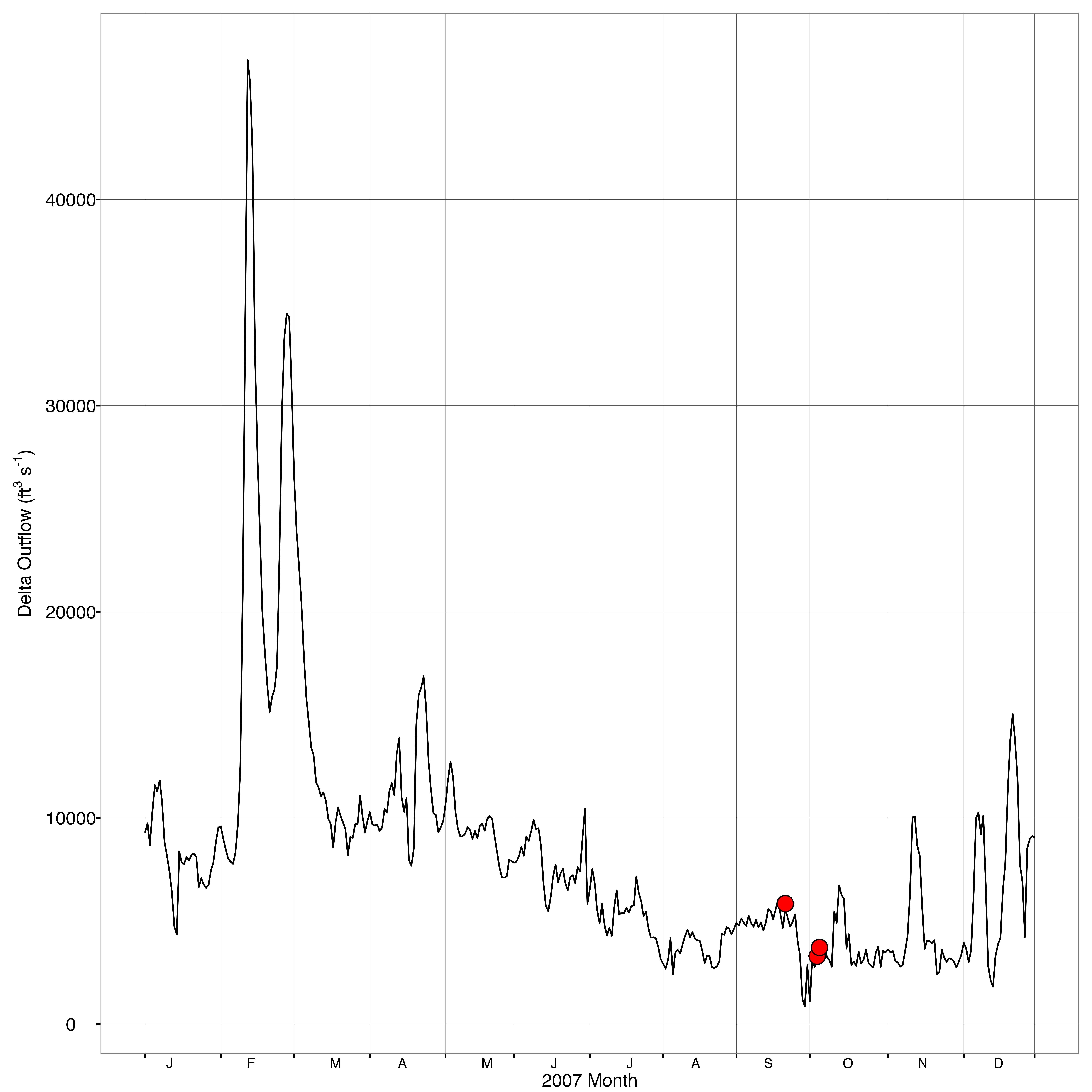


**Supplementary Figure A1. Delta outflow for the calendar year 2007.** Downloaded from the Dayflow database (maintained by the California Department of Water Resources). Sampling cruises in September and October 2007 are indicated by red dots. Sampling occurred following months of decreasing Delta outflows (i.e., lower river flows) during summer.


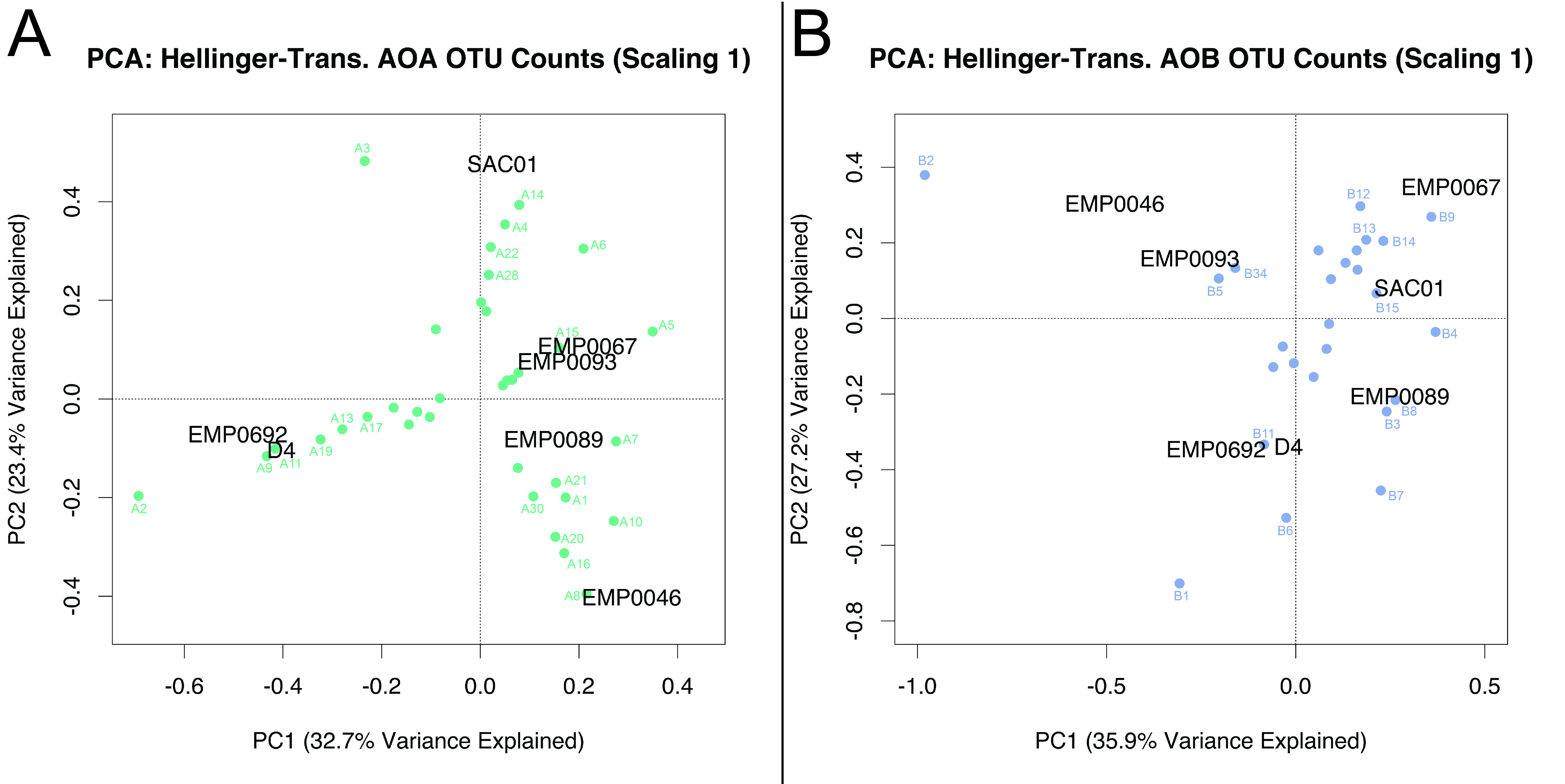


**Supplementary Figure A2. PCA biplots (scaling 1) of Hellinger-transformed *amoA* OTU counts.** (A) AOA and (B) AOB. Distance between stations on the biplot represents Euclidean distance in multidimensional space. Projections of a station onto an OTU vector indicate its position along that vector. Identity of OTUs is indicated, with those clustered near the center unnamed for legibility.


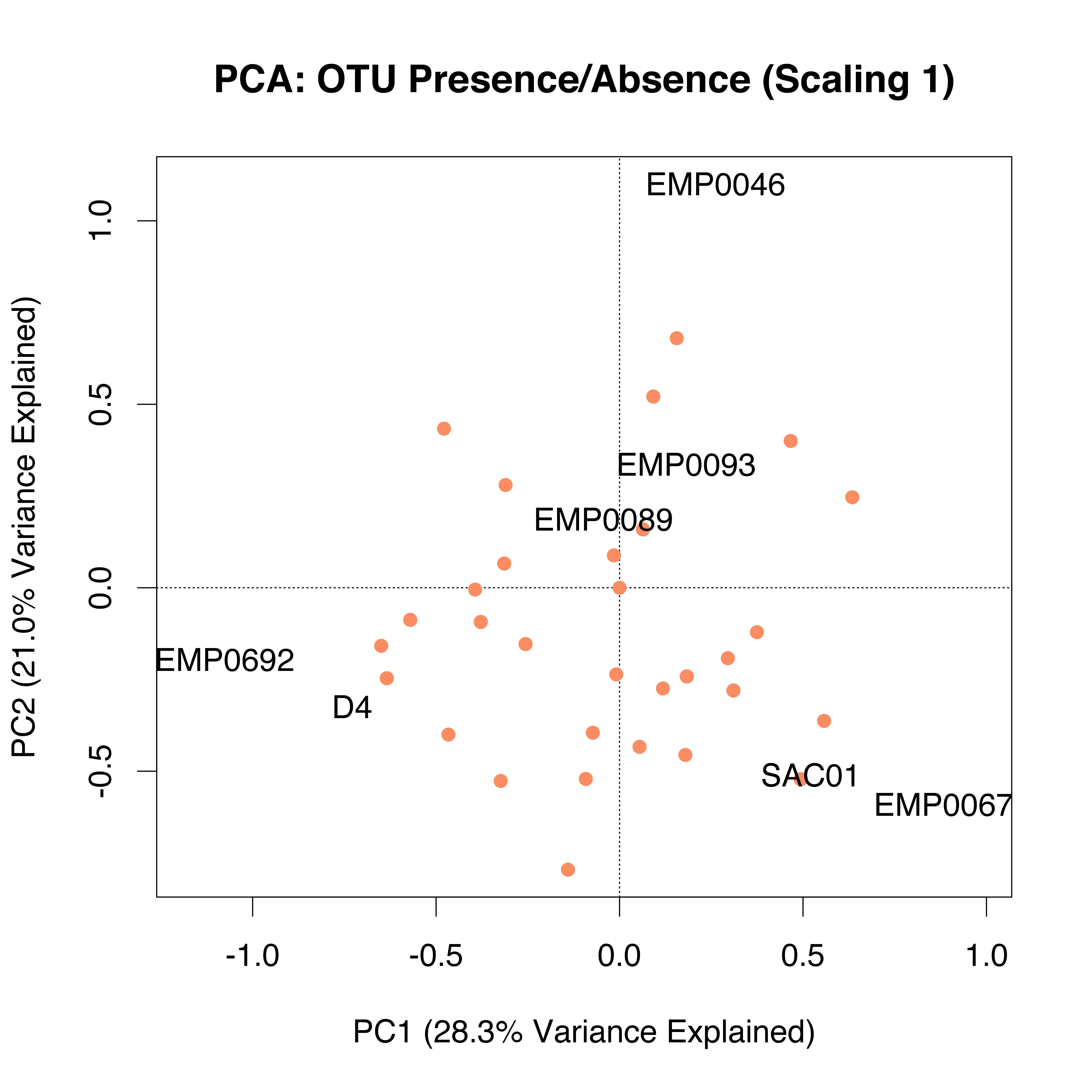


**Supplementary Figure A3. PCA biplot (scaling 1) of combined AOA and AOB *amoA* OTU presence/absence data in the Delta.** Distance between stations on the biplot represents Euclidean distance in multidimensional space. Projections of a station onto an orange OTU vector indicate its position along that vector.
